# Group Similarity Constraint Functional Brain Network Estimation for Mild Cognitive Impairment Classification

**DOI:** 10.3389/fnins.2020.00165

**Published:** 2020-03-10

**Authors:** Xin Gao, Xiaowen Xu, Xuyun Hua, Peijun Wang, Weikai Li, Rui Li

**Affiliations:** ^1^Shanghai Universal Medical Imaging Diagnostic Center, Shanghai, China; ^2^Tongji University School of Medicine, Tongji University, Shanghai, China; ^3^Department of Medical Imaging, Tongji Hospital, Shanghai, China; ^4^Yueyang Hospital of Integrated Chinese and Western Medicine, Shanghai, China; ^5^Shanghai University of Traditional Chinese Medicine, Shanghai, China; ^6^College of Computer Science and Technology, Nanjing University of Aeronautics and Astronautics, Nanjing, China; ^7^School of Mechanical and Precision Instrument Engineering, Xi’an University of Technology, Xi’an, China

**Keywords:** functional brain network, functional magnetic resonance imaging, group similarity constraint, mild cognitive impairment, Pearson’s correlation, partial correlation

## Abstract

Functional brain network (FBN) provides an effective biomarker for understanding brain activation patterns and a diagnostic criterion for neurodegenerative diseases detections. Unfortunately, it remains challenges to estimate the biologically meaningful or discriminative FBNs accurately, because of the poor quality of functional magnetic resonance imaging data or our limited understanding of human brain. In this study, a novel FBN estimation model based on group similarity prior was proposed. In particular, we extended the FBN estimation model to tensor form and incorporated the tensor trace-norm regularizer to formulate the group similarity constraint. To verify the proposed method, we conducted experiments on identifying mild cognitive impairments (MCIs) from normal controls (NCs) based on the estimated FBNs. Experimental results illustrated that our method is effective in modeling FBNs. Consequently, we achieved 91.97% classification accuracy, outperforming the state-of-the-art methods. The *post hoc* analysis further demonstrated that more biologically meaningful functional brain connections were obtained using our proposed method.

## Introduction

As a neurodegenerative disorder, Alzheimer’s disease (AD) is one of the most common causes of dementia (Wee et al., 2012). According to a recent report (Bain et al., 2008), the incidence of AD doublets every 5 years after age 60. AD seriously interferes with patients’ daily life, affects their memory and ability to communicate, and eventually causes their deaths. Unfortunately, there is no effective treatment for AD thus far. Hence, it is quite important to delay the onset and progression of AD during its early stages via pharmacological and behavioral interventions.

Mild cognitive impairment (MCI) is often considered as a critical time window and treatment period for the prediction or delaying the conversion in AD (Wee et al., 2012). In some recent statistical studies, nearly 10–15% patients with MCI develop probable AD each year (Grundman et al., 2004; Misra et al., 2009). The early detection and accurate diagnosis of MCI is considered a significant means of slowing AD progression (Alzheimer’s Association, 2017).

As a successful non-invasive technique, functional magnetic resonance imaging (fMRI) provides an effective method of measurement for revealing brain activities and patterns (Brunetti et al., 2006; Kevin et al., 2008; Jin et al., 2010). However, because spontaneous brain activity is random and asynchronous across subjects and scanners, it remains a challenge to identify MCI patients from normal controls (NC) by directly using the imaging information. Furthermore, high-order FBN-based statistical information provides new perspectives for discovering brain activity and connection patterns, thus improving our ability to understand brain information (Smith et al., 2011; Sporns, 2011; Wee et al., 2012; Stam, 2014; Rosa et al., 2015). In addition, various research has shown that the changing of functional brain networks are closely related to various neurological and psychological diseases such as AD (Supekar et al., 2008; Huang et al., 2009; Liu F. et al., 2012), MCI (Fan and Browndyke, 2010; Wee et al., 2012, 2014; Yu et al., 2016), autism spectrum disorder (ASD) (Theije et al., 2011; Gotts et al., 2012), Parkinson’s disease (PD) (Baggio et al., 2014), etc. All of these depend heavily on the quality of the final estimated FBN. Hence, improved FBN reliability is crucial to such estimates (Li et al., 2019a).

According to a FBN research review (Smith et al., 2011), correlation-based methods such as Pearson’s correlation (PC) (Li et al., 2017) and sparse representation (SR) (Lee et al., 2011; Zhou et al., 2014), are generally more sensitive than complex, high-order methods. However, due to the influence of noise in the observed data, correlation-based brain networks inevitably exhibit dense connections and thus contain substantial noise or false connections. One solution is to introduce sparse priors, as is done in the thresholding and SR (LASSO) methods. Actually, the topological structure of an FBN involves more than just sparsity (Sporns, 2011). Several studies (Lee et al., 2011; Qiao et al., 2016; Wee et al., 2016; Yu et al., 2016; Li et al., 2017, 2019b) have been focused on incorporating additional biological priors into FBNs to make them more discriminative. In practice, sparsity, modularity, group-sparsity, low-rank, and scale-free priors are commonly used (Lee et al., 2011; Qiao et al., 2016; Wee et al., 2016; Yu et al., 2016; Li et al., 2017). Moreover, priors can also be obtained from data quality (Li et al., 2019a) and other high-quality data (Li et al., 2019b). Note that most of the biological/data priors can be formulated into a regularized framework. This illustrates that a reliable FBN estimation model should both fit the data well and effectively encode brain organization priors (Qiao et al., 2016).

**FIGURE 1:**
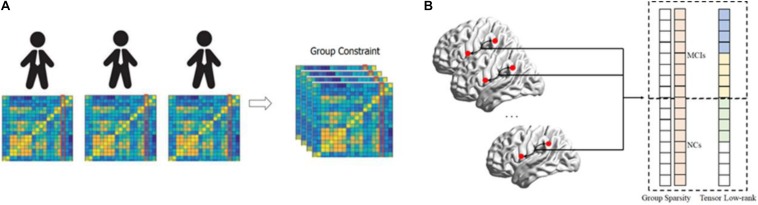
The motivation for the proposed tensor based FBN estimation model. **(A)** The estimated FBNs tend to have a group constraint. **(B)** The group lasso may easily lose discriminative features since it over-penalized or under-penalized connections from all subjects. In contrast, the tensor low-rank can effectively avoid this issue and thus naturally provide more discriminative connections.

Despite the advantages of existing FBN estimation methods, it is currently still an open field to estimate FBNs due to the complex of human brains and the poor quality of the observed data. In this paper, we focus on the group similarity prior of FBN (Wee et al., 2014), as shown in Figure 1A. In contrast, most current FBN estimation methods focus primarily on a single participant and rarely consider inter-group information from cross-participants, which result in different network topological structures across subjects. This performance inevitably makes comparisons between subjects difficult and thus can degrade the generalization performance of trained classifiers. Besides, the existing group constraint methods are mainly based primarily on the group sparsity penalty (i.e., *l*_2,1_-norm) to mitigate inter-subject variability (Wee et al., 2014; Yu et al., 2016). However, the specific information from individuals can be ignored, due to the additional *l*_2,1_-norm can often over-penalized or under-penalized connections of estimated FBN as shown in Figure 1B. In addition, some researchers have focused on group-fused multiple graphical-lasso schemes (Liang et al., 2016, 2018), which alleviating the issue of group sparsity constraints in some extent. As mentioned above, existing group-based FBN estimation approaches still have great potential.

In this paper, we use the regularization framework to incorporate the group similarity constraint into the FBN estimation model. In detail, we formulate the group similarity prior as a tensor low-rank (TLR) regularizer and incorporate it into the FBN estimation model. In addition, we further incorporate the sparse and tensor low-rank (STLR) for better FBN estimation. Since the low-rank is NP-hard, we optimize its upper limit (i.e., the trace norm penalization) for better calculation efficiency. In particular, we adopt Parallel factor analysis (PARAFAC) to calculate its eigenvalues (Liu X. et al., 2012) and design a proximal operator to estimate the FBN with the group similarity constraint. In the end, we incorporate the trace norm regularizer into the SR and PC models to create a simple test platform. To verify the proposed methods, we adopt an estimated FBN for MCI identification. In fact, the proposed method uses the group similarity constraints to shrink the FBN solution space, and thus can estimate more discriminative FBNs effectively. The highlights of this paper include:

1. We incorporate the group similarity constraint into the FBN estimation model using a low-rank regularizer. In addition, we further relax it into a trace norm regularizer and design an optimization algorithm to estimate FBNs with group similarity.
2. We use group similarity-based FBNs to separate MCIs from NCs. The experimental results show that the proposed scheme outperforms the baseline methods. Moreover, the proposed methods can provide more biologically meaningful connections than existing methods.
3. We provide an effective FBN estimation module useful in modeling the group similarity prior. The module is flexible enough to incorporate into other FBN estimation models. The experimental results show that the proposed module can effectively improve the MCI classification accuracies of the estimated FBNs.
4. We identified the most significant functional connections and the most discriminative brain regions using the proposed FBN estimation model. This analysis of functional connectivity and graph theory attributes can be used to discover biologically meaningful biomarkers and further elucidate the topological properties of a brain network that is experiencing MCI.

The remainder of this paper is organized as follows. In Section 2, we introduce our data preparation methodology. The proposed methods, i.e., the group similarity-based FBN estimation schemes, including the motivations, models, and algorithms are introduced. In Section 3, we evaluate the proposed methods using MCI identification experiments. A discussion and conclusions are presented in Sections 4 and 5, respectively.

## Materials and Methods

### Data Acquisition

For this study, we adopted publically available neuroimaging data from the Alzheimer’s Disease Neuroimaging Initiative (ADNI) database (Jack et al., 2010)^1^. ANDI was launched in 2003 by the National Institute on Aging, the National Institute of Biomedical Imaging and Bioengineering, the Food and Drug Administration, private pharmaceutical companies, and non-profit organizations. The initial goal of ADNI was to define biomarkers for use in clinical trials and to determine the best way to measure the treatment effects of AD therapeutics.

In particular, 137 participants including 68 MCIs and 69 NCs were adopted in this experiment, which was similar to a previous study (Zhou et al., 2018). The scanning parameters included: TR/TE = 3000/30 mm, flip angle = 80, imaging matrix = 64 × 64, 48 slices, 140 volumes, and voxel thickness = 3.3 mm. The SPM8 toolbox^2^ and DPARSFA (version 2.2) (Chao-Gan and Yu-Feng, 2010) were used to preprocess the fMRI data using the well-accepted pipelines. The preprocessing pipeline included removing the first 10 volumes, slice timing, realigning, normalizing, spatially smoothing, temporally detrending, regressing out covariates (ventricle and WM signals, as well as six head-motion parameters), and temporally filtering. We followed previous work (Chen et al., 2016, 2017) to alleviate head motion effects and artifacts and excluded subjects with more than 2.5 min (50 frames) of data with FD > 0.5 from further analysis (Power et al., 2012). Finally, we used the automated anatomical labeling (AAL) atlas (Tzourio-Mazoyer et al., 2002) to partition pre-processed blood-oxygen level dependent (BOLD) signals into 116 ROIs with 137 volumes. Finally, for *k*th participants, we put these volumes into a data matrix X^(*k*)^ ∈ *R*^*N*×*T*^(X for short). For more details, please refer to Zhou et al. (2018).

### Functional Brain Network Estimation

After obtaining fMRI data matrix **X** from the R-fMRI data, we performed FBN estimation. As mentioned above, correlation-based FBN estimation methods have been demonstrated to be more sensitive than some complex higher-order methods (Smith et al., 2011). Therefore, this paper focuses on correlation-based methods and adopts them as a baseline. In particular, we first defined the data matrix (i.e., the BOLD signal matrix) **X** ∈ *R*^*N*×*T*^, where *T* is the number of volumes and *N* is the number of ROIs. The fMRI time series associated with the ith ROI is represented by x*_i_* ∈ *R*^*T*^, *i*=1,⋯,*N*.

#### Correlation-Based Methods

As the simplest and most widely used FBN estimation schemes, PC-based FBN estimation methods account for a large proportion of FBN studies (Smith et al., 2013). The FBN edge weights **W** = (*W*_*ij*_) ∈ *R*^*N*×*N*^ can be calculated via PC as follows:

(1) $$W_{i\,j}\times \frac{{\left(x_{i}-{\bar{x}}_{i}\right)}^{T}\,\left(x_{j}-{\bar{x}}_{j}\right)}{\sqrt{{\left(x_{i}-{\bar{x}}_{i}\right)}^{T}\,\left(x_{i}-{\bar{x}}_{i}\right)}\,\sqrt{{\left(x_{j}-{\bar{x}}_{j}\right)}^{T}\,\left(x_{j}-{\bar{x}}_{j}\right)}},$$

In Eq. (1), $x_{i}-{\bar{x}}_{i}$ is a centralized counterpart of *x_i_*. Due to the effects of the noise in the fMRI data, PC always generates dense FBNs. Thus, a thresholding scheme is often used to make the PC-based FBNs sparse by filtering out the noisy or weak connections. The PC based FBN can be expressed as follows:

(2) Wi⁢j(n⁢e⁢w)×{Wi⁢j,Wi⁢j>t⁢h⁢r⁢e⁢s⁢h⁢o⁢l⁢d0,o⁢t⁢h⁢e⁢r⁢w⁢i⁢s⁢e,

where Wi⁢j(n⁢e⁢w) denotes the connection value between nodes *i* and *j* after thresholding.

When one compares PC measures to full-correlation cross ROIs, one notes that the interaction among multiple ROIs is neglected due to the cofounding effect. In contrast, a partial correlation is proposed by regressing out the confounding effects from other ROIs. However, the partial correlation-based methods can easily be ill-posed due to the singularity of the covariance matrix Σ×**X^*T*^X**. One simple solution is to incorporate an *l*_1_-norm regularizer into the partial correlation model (Lee et al., 2011), thus naturally incorporating the FBN sparsity prior (SR). The SR model is as follows:

(3) $$m\,i\,n_{W_{i\,j}}\,\sum_{i=1}^{n}{||x_{i}-\sum_{j\neq i}W_{i\,j}\,x_{j}||}^{2}+\lambda \,\sum_{j\neq i}\left|W_{i\,j}\right|,$$

The matrix form is proposed as follows:

$$m\,i\,n_{W}\,{||\text{X-XW}||}_{F}^{2}+\lambda \,{||W||}_{1}$$

(4) $$s.t.W_{i\,i}=0,\forall i=1,\cdots ,n,$$

Note that the *l*_1_-norm regularizer in Eq. (4) plays a key role in achieving a sparse, stable solution (Lee et al., 2011).

#### Regularization Framework for FBN Estimation

Based on the above description, both PC- and SR-based FBN estimation models can be summarized into the regularized FBN learning framework. We can naturally incorporate a regularized term and statistical information into the objective function in order to construct a new FBN estimation platform. Specifically, the platform can be formulated using a matrix-regularized learning framework as follows:

(5) $${\text{min}}_{W}\,f\,\left(X,W\right)+\lambda \,R\,\left(W\right),s.t.\text{W}\in △,$$

where *f* (**X**,**W**) models the FBN statistical information and *R*(**W**) is the regularization term used to incorporate FBN biological priors and stabilize solutions. In addition, some specific constraints such as symmetry or positive semi-definiteness may be included in △ to shrink the **W** search space. This provides an effective way of obtaining a better FBN. The λ is a hyper-parameter that controls the balance between the first (data-fitting) and second (regularization) terms.

Most of the recently proposed FBN estimation models (Higgins et al., 2018; Li et al., 2018; Wang et al., 2018; Zhou et al., 2018) can be unified under this regularized framework by re-designing the two terms in Eq. (5). Popular data-fitting terms include $||\text{W}-{\text{X}}^{T}\text{X}|{|}_{F}^{2}$ used in Eq. (2) and ${||\text{X–XW}||}_{F}^{2}$ used in Eq. (4), while popular regularization terms include l1-norm (Huang et al., 2010), trace norm, their combination (Qiao et al., 2016), etc.

#### Sparse and Low-Rank-Based FBN Estimation

Before we introduce the proposed method, we would like to review the sparse and low-rank-based (SLR) FBN estimation model briefly (Qiao et al., 2016). The sparsity and low-rank regularizers, i.e., the *l*_*1*_-norm and trace norm) causes sparse and similar connections across each brain region, naturally incorporating estimated FBN modularity priors. The SLR FBN estimation model is given as follows:

(6) $$m\,i\,n_{W}\,{||\text{X}-\text{XW}||}_{F}^{2}+\lambda \,{||W||}_{1}+\gamma \,{||W||}_{*},$$

where **X** is the BOLD signal data, ****W**** represents the estimated FBNs, λ||**W**||_1_ is the sparsity regularizer and γ||**W**||_*_ is the low-rank regularizer.

#### Group Sparsity-Based FBN Estimation

However, the abovementioned FBN estimation models are unable to deal with inter-subject variability problems because the FBN is estimated at an individual level, which easily causes different network topological structures across subjects To mitigate the effects of inter-subject variability, Wee et al. proposed a group-constrained sparse linear regression model (Wee et al., 2014) that applied the idea of joint feature selection in group-lassos to regression problems (Yuan and Lin, 2006). In particular, a group sparsity regularizer (GSR, i.e., *l*_2,1_-norm) was incorporated into the FBN estimation model. The GSR FBN estimation model is given as follows:

(7) $$m\,i\,n_{W_{j}}\,\sum_{k=1}^{N}{||{\text{X}}_{j}^{k}-{\text{X}}^{k}\,{\text{W}}_{j}^{k}||}_{F}^{2}+\lambda \,{||W||}_{2,1},$$

where **${\text{X}}_{j}^{k}$** is the BOLD signal of the *j*th ROI and *k*th participant, ****X^k^**** is the data matrix of *k* participants, **${\text{W}}_{j}^{K}$** represents the functional connections of the *j*th ROI and *k*th participant, and λ||**W**||_2,1_ is the group sparsity regularizer. Relative to the SR method, this minimizes inter-subject variability via an additional *l*_2_-norm regularizer across all subjects. However, these methods may penalize too much for estimated FBNs. For example, if a functional connection is removed from the MCIs but exists in NCs and the weight of this connection in the NCs is slightly larger than in MCIs, the GSR method tends to force removal of this connection from the NCs. In addition, if the number of NCs or the weight of this connection in the NCs is substantially larger than in the MCIs, the GSR method tends to force this connection to exist in the MCIs. Thus, a GSR can lose discriminative information from estimated FBNs, as shown in Figure 1B.

#### Methods

To incorporate group constraints easily and directly, we first extended the existing matrix regularization framework to tensor form as follows:

(8) $$m\,i\,n_{W}\,\sum_{\text{k}=1}^{n}f\,\left({\text{X}}^{\left(k\right)},{\text{W}}^{\left(k\right)}\right)-\lambda \,R\,\left(W\right)$$

where **X**^(*k*)^ ∈ *R*^*N*×*T*^ represents the input data of the *k*th participant., ROI is the number of predefined ROIs, *T* is the duration of data observation, and n is the number of participants. **W** ∈ *R*^*N*×*N*×*K*^ represents the estimated FBNs and **W**^(*k*)^ ∈ *R*^*N*×*N*^ represents the corresponding FBN of the *k*th participant. *K* is the number of participants. Obvious, in Eq. (8), **W** is 3-dimensional tensors. As with the matrix regularization framework, in Eq. (8), $\sum_{k=1}^{n}f\,\left({\text{X}}^{\left(k\right)},{\text{W}}^{\left(k\right)}\right)$ is the data-fitting term and *R*(**W**) is the regularization term in tensor format.

As shown in Figure 1, the abovementioned *l*_2,1_-norm penalty excessively punishes the estimated FBNs, which leads to interference across various groups in the data. To alleviate this issue, this paper uses the tensor regularization framework to relax the *l*_2,1_-norm penalty and naturally introduce the tensor low-rank (TLR) regularizer to formulate the group similarity prior. The proposed tensor low-rank-based FBN estimate can formulated as follows:

(9) $$m\,i\,n_{W}\,\sum_{k=1}^{K}f\,\left({\text{X}}^{\left(k\right)},{\text{W}}^{\left(k\right)}\right)+\lambda \,R\,{\left(W\right)}_{l\,o\,w\,r\,a\,n\,k}$$

For the regularized terms in Eq. (9), *R*(**W**)_lowrank_ indicates the rank of tensor **W**, which can be represented by number of non-zero elements in the eigenvalue of **W**. Unfortunately, the low-rank regularizer is non-convex with respect to W and is NP-hard to solve. Thus, we relax it to trace-norm ‖**W**‖_*_ and obtain the following optimization model:

(10) m⁢i⁢nW⁢∑k=1Kf⁢(X(k),W(k))+λ⁢||W||*

Here, we aim to capture the partial correlation of the observed fMRI data due to its empirical effectiveness. In particular, we adopted SR as a testing platform since the PC method suffers from cofounding effects. In particular, we used ${\left\|{\text{X}}_{\text{j}}^{k}-{\text{X}}^{k}{\text{W}}_{j}^{i}\right\|}_{F}^{2}$ as the data-fitting term to formulate the inverse covariance structure (i.e., partial correlation) in the data, and added a *l*_*1*_-norm penalty to encode the sparse priors, resulting in the following Sparse and Tensor Low-Rank (STLR) optimization model.

(11) m⁢i⁢nW⁢∑k=1K||Xk-Xk⁢Wk||F2+λ⁢||W||*+γ⁢||W||1

where λ and γ are hyper-parameters used to control the balance between the three terms in the objective function. It should also be noted that the data fitting term can be designed as ${||{\text{W}}^{k}-{\text{X}}^{\mathrm{kT}}\,{\text{X}}^{k}||}_{F}^{2}$ to capture full correlation statistics. In addition, when γ = 0, the proposed method reduces to the network learning model based on the traditional sparse regression FBN estimation method given in Eq. (4). As we can see in Eq. (11), when λ = 0, Eq. (8) will be reduced to TLR.

### Algorithm

Because the *l*_*1*_-norm and trace penalties exist, the proposed scheme is convex but non-differentiable. This leads to a non-trivial problem. Fortunately, several approaches have been proposed for dealing with such issues (Donoho and Elad, 2003; Meinshausen and Bühlmann, 2006; Tomioka and Sugiyama, 2009). In this paper, we use the proximal method (Combettes and Pesquet, 2011) to solve the proposed optimal FBN estimation model because of its simplicity and efficiency. The details are given as follows:

First, we address the STLR or TLR data-fitting term (i.e., $\sum_{k=1}^{n}{||{\text{X}}^{k}-{\text{X}}^{k}\,{\text{W}}^{i}||}_{F}^{2}$), whose gradient with respect to W^*k*^ is ∇Wk⁡f⁢(Xk,Wk)=XkT⁢Xk⁢Wk-XkT⁢Xk. For each iteration, we first update the W according to the gradient descent criterion:

(12) $${\text{W}}_{t}^{k}={\text{W}}_{t-1}^{k}-a_{t}\,\nabla f\,\left({\text{X}}^{k},{\text{W}}_{t-1}^{k}\right),$$

where *a_t_* denotes the gradient descent step size. The initial value of the step size *a_t_* was set to 0.001 and subsequently adaptively updated based on the line search scheme proposed by Nemirovski (Nesterov, 1983) using the SLEP toolbox^3^.

Second, we address the regularization term ||W||_1_. According to the definition of a proximal operator (Combettes and Pesquet, 2011), the proximal operator of ||W||_1_ is equivalent to the following soft thresholding operation on W,

(13) $${\text{prox}}_{\lambda ||\cdot |{|}_{1}}\left(W\right)=\text{[sign}\left({\text{W}}_{\text{ij}}\right)\times \max\left(W_{i\,j}-\lambda ,0\right)]{}_{n\times n},$$

Similarly, the proximal operator λ||W||_*_ corresponds to a shrinkage operation on the singular values of W as follows.

(14) proxλ||⋅||*⁢(W)=∑r=1Rmax⁢(λr-λ,0)⁢ai⁢r(1)⁢aj⁢r(2)⁢ak⁢r(3)

Here, *a*_*ir*_, *a*_*jr*_, *a*_*kr*_ is a vector in unit norm space and λ_*r*_ is the corresponding eigenvalue based on the parallel factor analysis (PARAFAC) (Liu X. et al., 2012). Then, the final algorithm is given in Table 1:

**TABLE 1:** The Algorithm for Estimating the FBN based on STLR/TLR.

Input: **X**,λ
Output: **W**
Initialize **W**
while *not converged*
**W**_*k*1_=*gradient*(**W**);
Wk⁢1=proxλ||⋅||*⁢(Wk);
**W**_*k*1_=prox*λ*||⋅||_1_(**W**_*k*_);
End


## Experiment

### Experimental Setting

**FIGURE 2:**
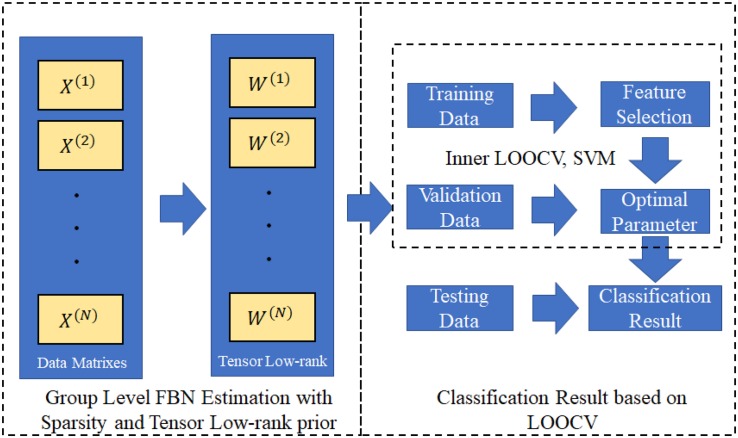
This flowchart shows the TLR/STLR implementation in group level FBN estimation and use of LOOCV for classification.

**FIGURE 3:**
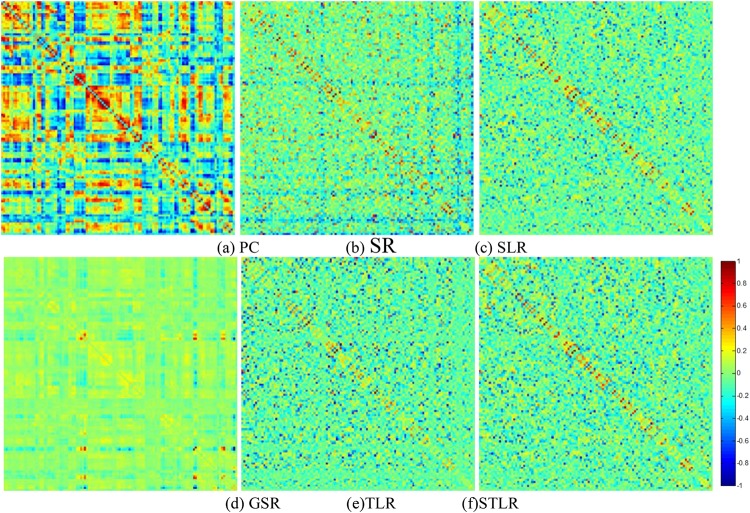
The FBN adjacency matrices of a certain subject, constructed by **(a)** PC, **(b)** SR, **(c)** SLR, **(d)** GSR, **(e)** TLR and (STLR).

After obtaining the FBNs of all subjects, the main task remaining was to use the aforementioned FBNs to train a classifier that could separate ASDs from NCs. Since the FBN matrix was symmetric, we used only its upper triangular elements as classification input features. Even so, the feature dimensions remained too high to train a classifier with good generalization due to the limited training sample availability in this study. Therefore, we performed feature filtering before classification training. Specifically, the classification pipeline included the following two main steps. A flow chart is given in Figure 2. First, we estimated FBNs for each individual using PC^4^, SR, SLR, GSR, TLR and STLR. The estimated FBNs are shown in Figure 3. After we obtained the estimated FBNs, we sought to determine how to use these connections to separate MCIs from NCs. It should be noted that both the feature selection and classifier design have large influences on the final accuracy (Wee et al., 2014). Because of this and because our focus was FBN estimation, we adopted the simplest feature selection method (*t*-test with *p*-value < 0.01) and used the most popular linear SVM classifier with default parameter C = 1 (Chang and Lin, 2007).

Due to the small sample size, we used the leave one out cross validation (LOOCV) strategy to verify method performance. In this strategy, only one subject was left out of testing while the others were used to train the models and determine optimal parameters. To choose optimal parameters, an inner LOO cross-validation was further conducted on the training data using a grid-search strategy. In the outer loop, we chosen the training and testing dataset to re-select feature and re-train the model by the selected parameters. More specifically, for the regularized parametersλ and γ, the candidate value range was [2^−5^,2^−4^,⋯,2^4^,2^5^]. For the hard threshold of PC_threshold_, we used 20 sparsity levels with a range of [5%, 10%,⋯,95%,100%]. For example, 90% means that 10% of the weak edges were filtered out of the FBN. In the outer loop, we used a training and testing dataset to re-select features and re-train the model based on the selected parameters.

### Network Visualization

For visual comparison of the FBNs constructed using the PC, SR, SLR, GSR, TLR and STLR methods, we first show the FBN adjacency matrices. *W* is shown as constructed via various methods in Figure 3. It can be observed from Figure 3 that the PC-based FBNs (i.e., Figure 3A) are quite different from the SR-based FBNs (i.e., Figures 3B–F) since they use a different data-fitting term [i.e., the first term in Eq. (5)]. Moreover, the topology of the FBN as estimated via SLR is similar to those produced by STLR and TLR because (1) both methods employ the same data-fitting term and (2) the low-rank and sparse regularity behind SLR (i.e., the trace norm in the matrix scheme) are based on the STLR result (i.e., the trace norm in the tensor scheme).

### MCI Identification

**FIGURE 4:**
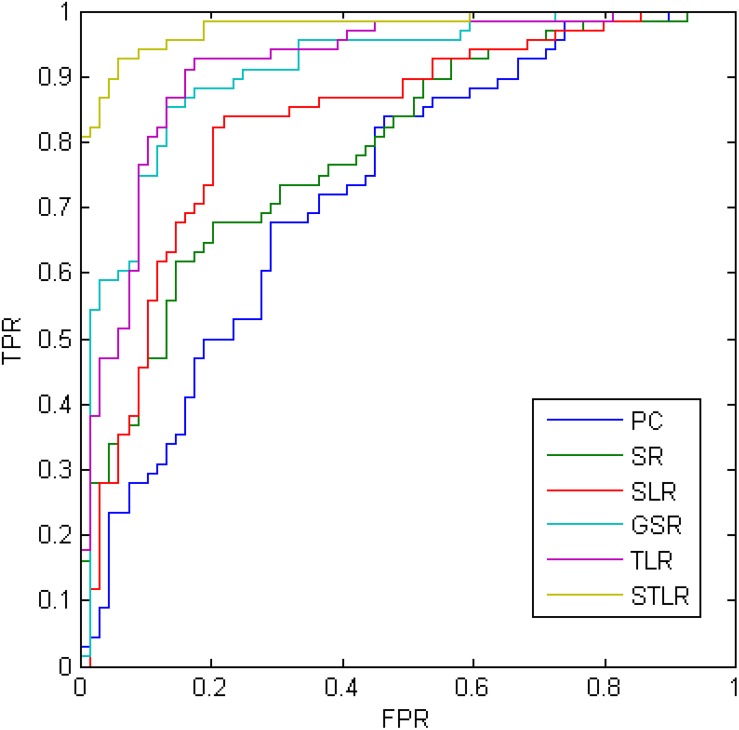
The ROC results of different methods.

The MCI versus NC classification results from the ADNI dataset are given in Table 2. The proposed STLR method achieved the best accuracy in this experiment. In addition, the SLR and GSR results are also provided in Figure 4 and Table 2.

**TABLE 2:** Classification performance of various FBN estimation methods on the ADNI dataset.

Method	Accuracy	Sensitivity	Specificity
PC	67.15	72.06	62.32
SR	78.10	79.41	76.81
SLR	80.29	80.88	79.71
GSR	83.21	88.24	78.26
TLR	85.40	86.96	83.82
STLR	91.97	92.65	91.30

A set of quantitative measurements, including accuracy, sensitivity and specificity, were used to evaluate the classification performances of six different methods (PC, SR, SLR, GSR, TLR and STLR). The mathematical definitions of these three measures follow:

$$A\,c\,c\,u\,r\,a\,c\,y=\frac{T\,r\,u\,e\,P\,o\,s\,t\,i\,v\,e+T\,r\,u\,e\,N\,e\,g\,a\,t\,i\,v\,e}{T\,r\,u\,e\,P\,o\,s\,t\,i\,v\,e+F\,a\,l\,s\,e\,P\,o\,s\,t\,i\,v\,e+T\,r\,u\,e\,N\,e\,g\,a\,t\,i\,v\,e+F\,a\,l\,s\,e\,N\,e\,g\,a\,t\,i\,v\,e},$$

(16) $$S\,e\,n\,s\,i\,t\,i\,v\,i\,t\,y=\frac{T\,r\,u\,e\,P\,o\,s\,t\,i\,v\,e}{T\,r\,u\,e\,P\,o\,s\,t\,i\,v\,e+F\,a\,l\,s\,e\,N\,e\,g\,a\,t\,i\,v\,e},$$

(17) $$S\,p\,e\,c\,i\,f\,i\,c\,i\,t\,y=\frac{T\,r\,u\,e\,N\,e\,g\,a\,t\,i\,v\,e}{T\,r\,u\,e\,N\,e\,g\,a\,t\,i\,v\,e+F\,a\,l\,s\,e\,P\,o\,s\,t\,i\,v\,e},$$

Here, *TruePositive* is the number of positive subjects that are correctly classified in the ASD identification task. Similarly, *TrueNegative*, *FalsePostive*, and *FalseNegative* are the quantities of their respective, corresponding subjects. In addition, the ROC of these methods is provided in Figure 4.

### Sensitivity to Network Model Parameters

**FIGURE 5:**
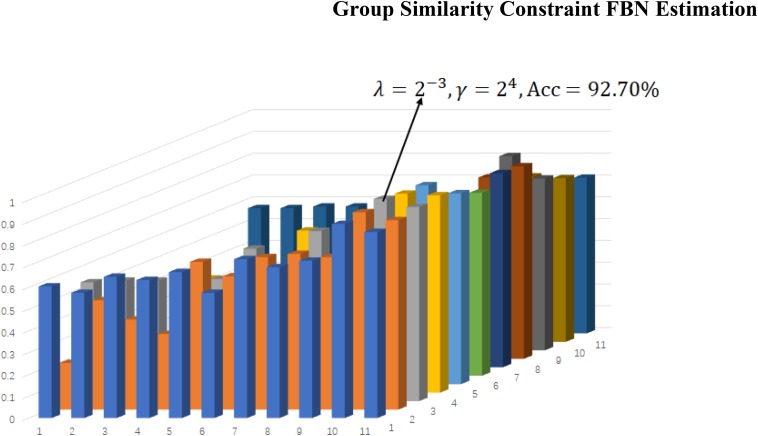
Classification accuracy based on the networks estimated by the proposed method with different regularized parametric values in the interval [2^−5^,2^5^]. The results are obtained by LOO test on all subjects.

Regardless of the FBN estimation method used, the classification accuracy is sensitive to various parameters (e.g., regularized SLR, GSR, TLR, and STLR parameters). Therefore, in our above classification experiments, we determine classification results with various parameters based on LOOCV. In Figure 5, we show the classification accuracies that correspond to various STLR parametric combinations. Figure 5 shows that we achieve the best accuracy (92.70%) with λ = 2^−3^ (for sparsity) and γ = 2^4^ (for tensor low-rank).

### Most Discriminant Brain Regions and Consensus Connections

**FIGURE 6:**
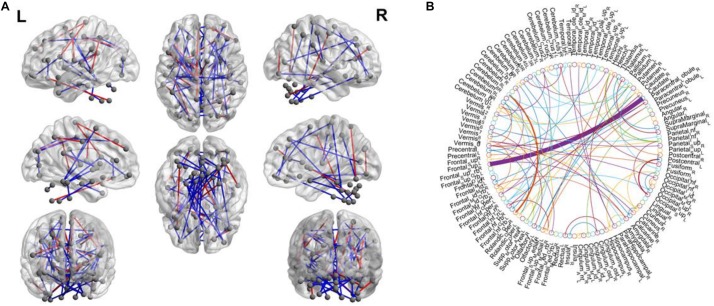
**(A)** The most significant functional connections mapped on the ICBM 152 template using the BrainNetViewer package (http://nitrc.org/projects/bnv/). The green and red lines represent connection weights that are decreased and increased in MCIs, respectively. **(B)** The consensus connections, selected via LOOCV, between MCI and NC for 116 AAL template ROIs. The arc thickness indicates the discriminative power of an edge, which is inversely proportional to the estimated *p*-values. This figure was created using a Matlab function, circularGraph, shared by Paul Kassebaum (http://www.mathworks.com/matlabcentral/fileexchange/48576-circulargraph).

As the selected connections in each inner loop might be different. We recorded all selected features during the training process. The statistics of the selected connections include a mean of 131.08 and variance 4.15. In addition, we further record the consensus connections for the classification model in each inner LOOCV loops. As mentioned above, we select the consensus connections with *p*-value < 0.01 in each loops, resulting in the 82 consensus connections shown in Figure 6. The thickness of an arc indicates the discriminative power of an edge, and is inversely proportional to the estimated *p*-values. The arc colors in Figure 6 are randomly generated to differentiate ROIs. In particular, there are 19 functional network connections that show decreased functional connectivity in patients with MCI.

In addition, we provide the most significant MCI and NC brain regions. In particular, the top 20 brain regions (without the cerebellum) with the largest number of discriminative connections (*p* < 0.01) are given in Table 3. The experimental results demonstrate that the brain regions that most discriminate between MCIs and NCs are distributed primarily in the thalamus, middle temporal gyrus, hippocampus, parahippocampal gyrus, inferior parietal (which corresponds to the subcortical network), Default mode network (DMN), dorsal attention network, and fronto-parietal task control network.

**TABLE 3:** The top 20 brain regions (without the cerebellum) with largest number of discriminative connections.

AAL Number	Corresponding brain regions	Sub-networks
77	Thalamus_L	Subcortical network
85	Temporal_Mid_L	Dorsal attention network
6	Frontal_Sup_Orb_R	Default mode network
9	Frontal_Mid_Orb_L	Default mode network
38	Hippocampus_R	Default mode network
39	ParaHippocampal_L	Default mode network
61	Parietal_Inf_L	Dorsal attention network
62	Parietal_Inf_R	Dorsal attention network
70	Paracentral_Lobule_R	Sensory/somatomotor hand
71	Caudate_L	Fronto-parietal task control
72	Caudate_R	Fronto-parietal task control
75	Pallidum_L	Subcortical network
11	Frontal_Inf_Oper_L	Executive control network
13	Frontal_Inf_Tri_L	Executive control network
24	Frontal_Sup_Medial_R	Fronto-parietal task control
42	Amygdala_R	Subcortical network
45	Cuneus_L	Visual network
47	Lingual_L	Default mode network
73	Putamen_L	Salience network
78	Thalamus_R	Subcortical network

### STLR on an Independent Dataset

To evaluate the purposed scheme further, we re-selected 50 independent participants (including 27 MCIs and 23 NCs) from the ADNI dataset to create an independent test dataset. Following the same preprocessing pipelines as mentioned above, the model was pre-trained on the aforementioned dataset with λ = 2^−3^ (for sparsity) and γ = 2^4^ (for tensor low-rank). Consequently, it achieved 86.00% accuracy, 91.30% sensitivity, and 85.19% specificity, which further demonstrated the effectiveness of the proposed method.

### Altered Functional Network Topological Properties in MCI Patients

Based on the FBN estimated via STLR with λ = 2^−3^ (for sparsity) and γ = 2^4^ (for tensor low-rank), several global graph theory metrics, including clustering coefficients (C_*p*_), the shortest path length (L_*p*_), the normalized clustering coefficient (γ), the normalized characteristic path length (λ), as well as small-world (σ) and global efficiencies (E_global_), were calculated to uncover the topological properties of functional networks in MCI and NC groups (Table 4). Moreover, we employed Modified Greedy strategy to calculate the modularity scores of the estimated FBNs (Newman, 2006). As expected, both groups fit γ = C_*p*_^real^ / C_*p*_^rand^ >1, λ = L_*p*_^real^ / L_*p*_^rand^ ≈1 and σ = γ/λ>1. Thus, the functional networks of MCI patients and NCs exhibit small-world topological attributes (Watts and Strogatz, 1998). This means that the brain networks of the two groups maintain complex, efficient neural architectures that optimize the balance between local specialization and global integration (Sporns and Zwi, 2004; Achard and Bullmore, 2007; Sporns, 2012). Further comparisons suggest that the small-world σ-values of MCI patients are lower than those of NCs, which indicates the disruption of the “economic small-world” (i.e., reductions in the effective segregation and integration of information in the brain network). Furthermore, we found the *C*_*p*_-values and modularities (*Q*-values) in MCI patients to be significantly lower than those in NC groups (*P* < 0.01). These changes in C_*p*_ and modularity suggest reduced local information processing network segregation in MCI patients. Although there is no significant difference between MCIs and NCs in L_*p*_ and E_global_, the lower values of these two global topological attributes in MCIs indicate decreased network integration.

**TABLE 4:** Statistical result of topological properties between MCIs and NCs.

	MCI	NC
Cp*	0.216 ± 0.016	0.262 ± 0.022
Lp	9.459 ± 2.956	9.886 ± 1.712
γ	1.147 ± 0.069	1.186 ± 0.099
λ	1.056 ± 0.018	1.062 ± 0.019
σ	1.086 ± 0.052	1.117 ± 0.088
Eglobal	0.032 ± 0.003	0.033 ± 0.002
Q*	0.202 ± 0.000	0.959 ± 0.000

* Significant with FDR (0.05).

Using the definition of “hubs” (Sporns, 2011), we identified hub nodes in MCI patients and NCs. As shown in Table 5, the common MCI and NC hub regions are located primarily in the bilateral superior temporal, bilateral heschl, right middle frontal, and left angular gyrus. Most are distributed in the DMN, auditory network, fronto-parietal task control network, and dorsal attention network. Moreover, it is notable that some hub nodes are present only in MCI patients but absent in NCs. These are the several hubs found in the right middle temporal and left middle frontal gyrus. In addition, there are some hub nodes found in HCs but not in MCI patients. They are located on the right inferior parietal and right middle frontal gyrus. These discriminative brain regions are distributed mainly in the DMN, fronto-parietal task control, and dorsal attention networks. Differences in subnetworks and corresponding brain regions play important roles in differential diagnosis of MCI relative to NC status.

**TABLE 5:** Hubs in MCI and NCs defined with the degree.

	AAL Number	Corresponding brain regions	Sub-networks
MCI	88	Temporal_Pole_Mid_R	Default mode network
	79	Heschl_L	Auditory network
	10	Frontal_Mid_Orb_R	Default mode network
	80	Heschl_R	Auditory network
	81	Temporal_Sup_L	Auditory network
	65	Angular_L	Default mode network
	87	Temporal_Pole_Mid_L	Default mode network
	9	Frontal_Mid_Orb_L	Fronto-parietal task control
	83	Temporal_Pole_Sup_L	Auditory network
	84	Temporal_Pole_Sup_R	Auditory network
NC	79	Heschl_L	Auditory network
	81	Temporal_Sup_L	Auditory network
	65	Angular_L	Default mode network
	66	Angular_R	Default mode network
	87	Temporal_Pole_Mid_L	Default mode network
	10	Frontal_Mid_Orb_R	Default mode network
	80	Heschl_R	Auditory network
	62	Parietal_Inf_R	Dorsal attention network
	84	Temporal_Pole_Sup_R	Auditory network
	83	Temporal_Pole_Sup_L	Auditory network

*AAL, the automated anatomical labeling atlas.*

## Discussion

The human brain is one of the most complex systems in the world. To ensure efficient brain information interactions, the FBN should have more “structures” than sparsity (Smith et al., 2011; Sporns, 2011). In this work, we incorporated a tensor low-rank regularizer to model the group similarity priors of the estimated FBNs. The MCI versus NC classification capabilities of the proposed models were verified using the ADNI dataset. Based on the results, we give the following brief discussion.

1. STLR-based methods were more accurate than baseline and state-of-art methods on our dataset. One possible reason is that the STLR scheme naturally incorporates additional information from inter-group subjects, and thus can produce clearer or more discriminative FBNs. It should also be noted that the proposed scheme is a flexible module. In addition to using SR-based models, it can be easily adopted using other FBN estimation models such as PC-based, Bayesian, or Granger causal-based networks. Also, we can incorporate other biological group priors into the tensor-based FBN estimation models.
2. The most discriminative functional connections and the corresponding predominating brain regions were discussed. By projecting brain regions with significant brain network functional connectivity differences and graph theory metrics to subnetworks, we found that the differences between MCI patients and NCs were distributed mainly in the DMN, dorsal attention, frontoparietal task, executive control, and auditory networks. Of these, the DMN had the most significant discriminative ability. Changes in these subnetworks were consistent with the results of previous cognitive function studies such as those on spatial attention (Rolle et al., 2017), executive function (Liao et al., 2019), and auditory function (Bi et al., 2018) that reference subnetworks in MCI patients. Moreover, the DMN has been regarded as the core part of a functional center (Liu et al., 2019) that is involved in episodic memory and is thought to be the major cognitive domain impaired during early-stage AD (Eyler et al., 2019). That the DMN contains the most distinguishing information for MCI identification was verified using our proposed methods. Furthermore, in our study, we located the predominant brain regions (i.e., the thalamus, middle temporal gyrus, hippocampus, parahippocampal gyrus, and inferior parietal and middle frontal gyrus) for MCI diagnosis.
3. Brain network patterns are altered in MCI patients. Our study found that the global topological properties of MCI patients and NCs fit the small-world attribute. That is, the brain networks of MCI and NC groups conform to “economic small-world” classification, which uses rapid, real-time information processing across separate brain regions to maximize efficiency with minimal cost and to render resilience against pathological attacks (Sporns and Zwi, 2004; Sporns, 2012; Liao et al., 2017). Statistical analysis suggested that the value of small-world σ was lower in MCI patients than in NCs, which indicated disruption of brain network integration and segregation. This MCI small-world result is consistent with previous research (Yu et al., 2018). Moreover, the significantly decreased C_*p*_ and modularity values noted in MCI patients further verified the reduction in brain network functional segregation. Lower *C*_*p*_- and *Q*-values suggest less concentrated clustering of local connections and a weaker capacity for specialized processing within densely interconnected groups of brain regions in MCI patients (Rubinov and Sporns, 2010).

However, since the proposed scheme is a simple attempt to model the group similarity prior, there are several limitations in the proposed methods that should be improved upon via future work.

1. In this paper, we provide only simple verification to validate the effectiveness of the TL scheme and do not consider other factors (e.g., Atlas selection and data preprocessing). Therefore, we simply adopt the commonly used AAL atlas to define ROI. In the future, we would like to consider using a functional template (e.g., Power264) to alleviate this issue.
2. In this paper, we use only the tensor low-rank module to formulate the group similarity prior. In fact, the brain has a highly complex structure, and group similarity can be formulated into other formats. Therefore, we will use more abundant biological/physical priors to construct appropriate regular terms and further improve the current group-constraint model in future studies.
3. The global graph theory metrics (i.e., C_*p*_, Lp, and small-world) were areas of focus in our study. However, nodal and other graph theory metrics could also be used to describe the complex topological mechanisms of brain networks. In future research, more graph theory metrics, such as the nodal shortest path length, local efficiency, and participant coefficient of modularity may be used to elaborate upon more specific local brain network topological properties.

## Conclusion

Human brain patterns still need deep exploration. Thus, providing better brain descriptions remains challenging and meaningful. Inspired by the group similarity priors, we introduced the tensor based FBN estimation scheme. In particular, we proposed TLR and STLR to estimated FBN. More specially, we used the PARAFAC decomposition to capture FBNs with low-rank topologies. Finally, we applied the estimated FBNs to classification. The results illustrate that the introduction of the group similarity constraint can effectively improve baseline method performance. The *post hoc* analysis further showed that more biologically meaningful functional brain connections were obtained by incorporating the group similarity prior.

## Data Availability Statement

Publicly available datasets were analyzed in this study. This data can be found here: http://adni.loni.ucla.edu.

## Author Contributions

All authors developed the proposed algorithm and architecture. WL and RL designed the evaluation experiments. XG, PW, and XH preprocessed the fMRI. XX and XH analyzed and interpreted the results of the data. All authors contributed to preparation of the article, figures, and charts.

## Conflict of Interest

The authors declare that the research was conducted in the absence of any commercial or financial relationships that could be construed as a potential conflict of interest.

## Footnotes

1 http://adni.loni.ucla.edu

2 http://www.fil.ion.ucl.ac.uk/spm/

3 http://yelabs.net/software/SLEP/

4 In order to improve PC flexibility and conduct fair comparisons, we introduced a hard-thresholding PC parameter by reducing a portion of weak connections.
